# Non-invasive brain stimulation for chronic low back pain: current techniques and future perspectives

**DOI:** 10.3389/fnhum.2026.1760097

**Published:** 2026-02-11

**Authors:** Jianpeng Zou, Shijie Hao, Gang Li

**Affiliations:** 1 First College of Clinical Medicine, Shandong University of Traditional Chinese Medicine, Jinan, Shandong, China; 2 Department of Rehabilitation, Affiliated Hospital of Shandong University of Traditional Chinese Medicine, Jinan, Shandong, China; 3 College of Rehabilitation Medicine, Shandong University of Traditional Chinese Medicine, Jinan, China; 4 Orthopaedic Microsurgery, Affiliated Hospital of Shandong University of Traditional Chinese Medicine, Jinan, Shandong, China

**Keywords:** chronic low back pain, neuroplasticity, non-invasive brain stimulation, transcranial magnetic stimulation, transcranial direct current stimulation, transcranial focused ultrasound

## Abstract

Chronic low back pain has a high incidence rate and poses a threat to human physical and mental health. As the disease progresses over time, patients with chronic low back pain may exhibit corresponding clinical symptoms not only in localized back pain and functional limitations but also in movement patterns, psychological states, and cognitive aspects. As research continues to advance, maladaptive changes in the brains of patients with chronic low back pain have drawn the attention of scholars. Under sustained pathological stimulation, structural and functional alterations may occur in brain regions such as the sensorimotor cortex, prefrontal cortex, and cerebellum. Recently, non-invasive brain stimulation techniques have gained widespread clinical application and hold significant therapeutic value in the treatment of chronic low back pain. This paper outlines the targets and mechanisms of non-invasive brain stimulation techniques for treating chronic low back pain. It summarizes the current clinical applications of transcranial magnetic stimulation and transcranial direct current stimulation in patients with chronic low back pain, and explores the prospects of non-invasive brain stimulation for managing this condition. The aim is to introduce novel approaches to rehabilitation therapy for chronic low back pain and provide a solid foundation for future research directions.

## 1 Introduction

Chronic low back pain (CLBP) is one of the major health issues affecting humanity worldwide (GBD 2021 Low Back Pain Collaborators, 2023). CLBP persists for at least 12 weeks and is often accompanied by pain in one or both lower limbs. It can adversely affect patients’ physical, psychological, and cognitive-behavioral aspects, making it one of the leading causes of disability (Zhu et al., 2024). The etiology of CLBP is complex. Based on causative factors, it can be categorized into two main types: chronic specific low back pain and chronic nonspecific low back pain. Chronic nonspecific low back pain accounts for approximately 90% of cases (Medrano-Escalada et al., 2022). Decreased spinal stability, poor postural control, alterations in brain structure and function, inflammatory factor stimulation, and psychosocial factors are among the pathological mechanisms underlying CLBP. Traditional views hold that local injuries or related changes determine functional impairments associated with CLBP; thus, treatment focuses on the lumbar spine and its associated structures. With the increasing depth of research, growing evidence indicates that patients with CLBP exhibit neuroplastic changes significantly associated with chronic pain, such as alterations in brain structure, function, and metabolism. Clinical studies have revealed that brain regions associated with pain perception, motor function, emotional processing, and cognitive function exhibit abnormal changes in patients with CLBP (Chen et al., 2025). These maladaptive structural and functional alterations may play a role in perpetuating pain (Ehrenbrusthoff et al., 2022). Therefore, based on the focus on local structural and biomechanical factors, targeting the regulation of neural plasticity has become a key research direction in the treatment of CLBP (Kandić et al., 2021; Zou and Hao, 2024). Neuroplasticity is the nervous system’s capacity to respond to internal and external stimuli such as physiological changes, pathological damage, and environmental alterations. Its essence lies in the brain’s self-regulatory mechanism, which adapts through structural and functional changes to cope with chronic stress, injury, and other challenges. Neuroplasticity is essential for normal brain function and adaptation, but in chronic pain conditions, maladaptive plasticity may occur, amplifying and sustaining pain signals while triggering various physical and psychological symptoms. Therefore, neuromodulation techniques targeting neural plasticity are increasingly becoming important therapeutic approaches for chronic pain (Liu et al., 2025; Jayathilake et al., 2025).

## 2 Non-invasive brain stimulation techniques for CLBP

Non-invasive brain stimulation (NIBS) is developed based on the principle of neuroplasticity. It promotes the reorganization of neural structures and functions by applying electrical, magnetic, acoustic, or light stimuli to restore or optimize brain function. NIBS has been widely applied in the treatment of neurological and psychiatric disorders due to its significant role in regulating brain activity and improving cognitive and motor functions. In recent years, the application of NIBS in treating chronic pain has attracted the attention of scholars. NIBS techniques can modulate the excitability of brain-related cortical areas and neural networks involved in pain processing, making them promising intervention methods for chronic pain (Xiong et al., 2022; O'Connell et al., 2018). In the field of chronic pain, the primary motor cortex (M1) and prefrontal cortex are the most commonly targeted regions for brain stimulation (Kong et al., 2024). The application of NIBS in CLBP also typically focuses on these two areas. Currently, among various NIBS techniques, transcranial magnetic stimulation (TMS) and transcranial direct current stimulation (tDCS) have become the most widely used interventions for treating CLBP. NIBS has been demonstrated to improve pain, enhance athletic performance, regulate psychological states, and exert numerous other potential effects. It can also effectively activate brain regions such as the primary motor cortex, thereby promoting the efficacy of other rehabilitation measures (Alcon and Wang-Price, 2022; Teixeira et al., 2020).

### 2.1 Therapeutic targets

#### 2.1.1 The primary motor cortex

As the disease progresses, changes in brain structures and functional activity associated with CLBP patients may affect spinal motor control (Gilliam et al., 2024). On one hand, persistent pain stimulation and pathological alterations in peripheral receptors of the lumbar region can lead to reorganization and functional changes in the sensory cortex. This affects the lumbar sensory-motor feedback mechanism, influencing descending motor control through associated neural circuits and resulting in reduced spinal stability. On the other hand, regional alterations in brain activity induced by pain may further disrupt information encoding, causing motor coordination disorders that impair the function of paraspinal muscles and compromise spinal stability (Zhang et al., 2024; Massé-Alarie et al., 2024). Research has found reduced excitability in the motor cortex of patients with CLBP (Corti et al., 2022). Another clinical study employed transcranial magnetic stimulation to map the cortical representation of paraspinal muscles in CLBP patients, revealing anterior displacement and lateral reorganization of the center of gravity of the cortical maps in all subjects. The above studies suggest that reorganization of paraspinal muscle cortical representations may be associated with the pathology of CLBP (Saraiepour et al., 2023). The establishment of spinal stability requires precise motor planning and sensory information processing and integration at the central level. Changes above the central nervous system may lead to maladaptive alterations in motor output, potentially contributing to the development of CLBP. The primary motor cortex (M1) is responsible for the generation and execution of movement, playing a crucial role in establishing spinal stability. Studies indicate that reorganization of the cortical region representing paraspinal muscles in primary motor cortex (M1) leads to altered activation of muscles such as the lumbar multifidus and erector spinae, which in turn is closely associated with impaired spinal motor control and CLBP (Massé-Alarie et al., 2016). Other scholars propose that reduced excitability in the primary motor cortex (M1) of CLBP patients leads to diminished neural drive to the multifidus muscles, constituting a key pathophysiological mechanism of CLBP (Silfies et al., 2017; Abd-Elsayed et al., 2025). Consequently, the primary motor cortex (M1) has become the most frequently targeted region for non-invasive neuromodulation in CLBP.

#### 2.1.2 The prefrontal cortex

CLBP can induce changes in brain regions associated with sensation, cognition, attention, and emotional processing, leading to alterations in cognitive and affective behaviors (Marshall et al., 2017; Oosterman et al., 2012). Research indicates that approximately one-third of chronic pain patients exhibit cognitive deficits affecting attention, learning, memory, and other functions (Moriarty et al., 2017). Individuals with CLBP face a higher risk of cognitive decline due to alterations in cerebral cortex and neural network activity, including gray matter atrophy, glial cell activation, and neuroinflammation (Zhou et al., 2022). Additionally, pain catastrophizing, kinesiophobia, anxiety, and depression frequently occur in patients with CLBP, creating a vicious cycle that negatively impacts health status and rehabilitation outcomes (de Alencar et al., 2025; Cao et al., 2025). The prefrontal cortex (PFC) is involved in motivation, decision-making, and pain regulation. Therefore, cognitive and emotional behavioral changes in patients with CLBP are closely associated with PFC (Sunavsky et al., 2025). Within the prefrontal cortex region, the medial prefrontal cortex (mPFC) serves as a key brain area mediating the interaction between pain, emotional dysregulation, and anti-injury sensations (Kummer et al., 2020). While the dorsolateral prefrontal cortex (DLPFC) is considered an interface between three major brain networks: the resting-state default mode network, the salience network, and the fronto-parietal network, participating in pain, cognitive, and emotional processing. Research indicates that during chronic pain, the DLPFC exhibits structural and functional alterations, including volume reduction, altered activation patterns, and diminished connectivity with other brain regions (Seminowicz and Moayedi, 2017). Additional studies reveal increased activation in the DLPFC of patients with CLBP (Zeng et al., 2023). Zhao et al. (2025) observed alterations in spontaneous brain activity and functional connectivity in patients with CLBP who also had comorbid depression. Their findings revealed significant intergroup differences in right DLPFC regional homogeneity (ReHo) values among CLBP patients with depression compared to both depression-free and healthy control groups. Additionally, changes in functional connectivity were observed between the DLPFC and the cerebellum, as well as between the DLPFC and the orbitofrontal cortex. Based on the above relevant studies, the DLPFC has emerged as another commonly targeted region for modulating pain through brain pathways (Ueno et al., 2025).

### 2.2 Mechanism of action

#### 2.2.1 Modulating cortical excitability

NIBS exerts its effects by acting on specific brain functional areas to influence cortical excitability and synaptic efficacy (Bhattacharya et al., 2025). TMS generates induced currents through brief, rapidly changing magnetic fields applied to specific brain regions, thereby exerting neuromodulatory effects (Cotovio et al., 2023; Iglesias, 2020). TMS significantly upregulates proteins crucial for synaptic plasticity, such as brain-derived neurotrophic factor (BDNF), Tyrosine Kinase receptor (TrkB), N-methyl-D-aspartate receptor 1, and phosphorylated cAMP-response element binding protein (CREB) (Liu et al., 2026). TMS modulates synaptic plasticity, inducing long-term potentiation (LTP) and long-term depression (LTD) effects, thereby regulating cortical functional activity and neuronal excitability. The basis of these changes lies in alterations of glutamatergic and GABA neurotransmission. Changes in neural transmission signals, along with alterations in the synthesis and release of relevant neurotransmitters, activation of associated receptor activity, and modifications in the properties of related ion channels, subsequently influence dendritic spine formation, synapse generation, and renewal (Popovic and Dragic, 2025). Low-frequency repetitive transcranial magnetic stimulation (rTMS) (≤5 Hz) induces inhibitory effects, leading to cortical activity suppression and reduced neuronal excitability, whereas high-frequency rTMS (>5 Hz) enhances cortical activity, primarily exerting excitatory effects. tDCS delivers a constant, low-intensity electrical current through electrodes, modulating sodium and calcium ion channels to alter resting membrane potential and thereby regulate neuronal excitability. Anodal transcranial direct current stimulation (a-tDCS) increases excitatory activity by lowering the resting membrane potential of neurons, while cathodal transcranial direct current stimulation (c-tDCS) inhibits activity in the relevant brain regions by hyperpolarizing the resting membrane potential. a-tDCS enhances cortical excitability by promoting calcium influx through N-methyl-D-aspartate (NMDA) receptors, thereby inducing long-term potentiation (Stagg and Nitsche, 2011). NIBS can also exert neuromodulatory effects beyond the targeted cortical regions. Activation of localized functional brain areas can trigger the propagation of action potentials, thereby producing therapeutic effects on interconnected brain regions and even entire brain networks.

#### 2.2.2 Modulating neuroinflammation

Neuroinflammation may be a common phenomenon among patients with chronic pain, and neuroimmunomodulation warrants more active exploration as a potential therapeutic strategy for chronic pain (Loggia, 2024). Activation of glial cells in the sensory-motor cortex (S1, M1), along with alterations in synaptic transmission and neuronal excitability, may represent key mechanisms sustaining nociceptive processes in patients with CLBP. The increased activation of neuroinflammation in the somatosensory-motor regions (S1/M1) of patients with CLBP has been identified as a key factor underlying their somatosensory alterations and central sensitization. Furthermore, this neuroinflammatory activation shows a positive correlation with indicators such as pain sensitivity, insomnia, depression, and functional disability (Shraim et al., 2024). NIBS can activate brain immune cells such as microglia and astrocytes, enhancing neuroprotection and promoting brain functional recovery through cytokines and chemokines (Demir et al., 2026). rTMS exerts neuroprotective effects by modulating microglial activation and enhancing neuroplasticity processes (d'Errico et al., 2025). Some researchers suggest that rTMS alleviates pain and depressive symptoms in musculoskeletal pain patients by regulating neuroinflammation, transporter activity, and ion channel modulation, thereby improving quality of life (Liang et al., 2025; Li T. et al., 2025).

### 2.3 Clinical applications

#### 2.3.1 TMS

TMS directly acts upon the brain, influencing its functional circuits, and holds potential benefits in promoting improved clinical outcomes and neuroadaptive changes. TMS applied to the primary motor cortex (M1) region can improve motor control through central-peripheral motor pathways and induce changes in brain areas associated with pain (Yang and Chang, 2020). Studies indicate that rTMS, by acting on the primary motor cortex (M1) region, can modulate the concentration of neurotransmitters such as gamma-aminobutyric acid (GABA), regulate cortical excitability, and exert analgesic effects (Hoogendam et al., 2010). Li et al. (2024) investigated the efficacy of rTMS targeting the primary motor cortex M1 combined with sling exercise for patients with CLBP. After a 2-week intervention, the combined group demonstrated significant improvements in multifidus muscle activation, as measured by the Numerical Pain Rating Scale and the Oswestry Disability Index (ODI). Compared to rTMS alone, the combined approach showed potential clinical value in influencing motor cortex plasticity and enhancing trunk muscle activation. In addition to activating localized brain regions, rTMS can also exert therapeutic effects by modulating functional connectivity within relevant brain networks through the synaptic propagation of action potentials. Mahboubeh Masoumbeigi et al. analyzed the analgesic effects of rTMS on patients with nonspecific CLBP using resting-state functional magnetic resonance imaging. Fifteen patients with nonspecific CLBP were selected, with the primary motor cortex as the target area and a stimulation frequency of 20 Hz. Results showed significantly reduced pain intensity post-rTMS compared to pre-stimulation (*p* < 0.05). Additionally, increased amplitude of low-frequency fluctuation (ALFF) was observed in mPFC following rTMS, while decreased ALFF was noted in the insula (INS), thalamus (THA), and supplementary motor area (SMA). Findings indicate spontaneous brain activity correlates with pain intensity in nonspecific CLBP patients. The analgesic mechanism by which rTMS reduces pain intensity in nonspecific CLBP may involve alterations in spontaneous neural activity across distinct brain regions, including the insula, thalamus, mPFC, precuneus, and supplementary motor area (Masoumbeigi et al., 2024). Li et al. investigated the effects of combined rTMS and repetitive peripheral magnetic stimulation on pain in patients with chronic nonspecific low back pain. rTMS targeted the contralateral primary motor cortex (M1) to the painful side at a frequency of 20 Hz and intensity of 90% RMT. After 2 weeks of treatment, both groups showed significant reductions in Visual Analogue Scale (VAS) and ODI scores compared to pre-treatment levels (both *p* < 0.05). Functional near-infrared spectroscopy (fNIRS) revealed that post-treatment, compared to the control group, the experimental group demonstrated significantly increased activation in the somatosensory association cortex (SAC) region, along with improved functional connectivity in brain regions including the SAC and primary motor cortex. These findings suggest that closed-loop magnetic stimulation therapy can significantly alleviate pain in patients with chronic nonspecific low back pain and remodel relevant brain regions, warranting clinical application and further research (Li C. et al., 2025).

Additionally, TMS applied to DLPFC can improve pain and cognitive behavioral abnormalities by modulating relevant neural network connectivity. Given the strong association between chronic pain and major depressive disorder, rTMS targeting the dorsolateral prefrontal cortex has demonstrated efficacy in treating major depressive disorder, and patients with CLBP may also benefit from this approach (Afshar et al., 2024). Yang et al. investigated the effects and potential mechanisms of intermittent theta burst stimulation (iTBS) applied to DLPFC on pain in patients with CLBP. Forty patients were randomly assigned to two groups: one receiving iTBS combined with core stability training, and the other receiving sham iTBS combined with core stability training. Resting-state functional magnetic resonance imaging (fMRI) scans were performed before and after intervention. Results revealed that iTBS combined with core stability training significantly improved pain and related cognitive-behavioral abnormalities. Enhanced functional connectivity between the DLPFC and the cerebellum, as well as the occipital gyrus, emerged as a potential mechanism (Yang et al., 2025). Theta burst stimulation (TBS), as a form of TMS stimulation, primarily encompasses two modes: iTBS and continuous TBS. The former primarily exerts an excitatory effect, while the latter primarily exerts an inhibitory effect (Wang et al., 2025). iTBS, as a novel rTMS modality, demonstrates stronger and more enduring effects on cortical excitability in chronic pain patients compared to conventional rTMS.

#### 2.3.2 tDCS

a-tDCS targeting the primary motor cortex significantly enhances cortical activation and improves postural control in patients with CLBP (Li Y. et al., 2025). Jafarzadeh A et al. found that a-tDCS targeting the primary motor cortex (M1) combined with posture training (a-tDCS) targeting the primary motor cortex (M1) combined with posture training significantly improved balance, postural stability, and pain in patients with CLBP. Compared to sham a-tDCS combined with posture training or posture training alone, statistically significant improvements were observed in postural stability measures, Berg Balance Scale scores, and VAS scores (Jafarzadeh et al., 2019). Hejazi et al. (2025) found that a-tDCS combined with sensorimotor training (SMT) increased excitability in sensory and motor cortex regions, improved anticipatory and compensatory postural control strategies, and alleviated related symptoms. a-tDCS targeting the prefrontal cortex can also modulate cortical excitability and improve postural stability. Masoudi et al. (2024) found that targeting DLPFC with both a-tDCS and c-tDCS improved postural control during cognitive posture tasks in patients with CLBP complicated by high pain anxiety. The improvement was more pronounced in the a-tDCS group. Ehsani et al. (2023) also found that anodal and cathodal tDCS targeting the left DLPFC improved postural stability during environmental challenges in patients with CLBP experiencing high fear of pain, with a-tDCS appearing to be more effective. Alcon et al. (2024) found that a-tDCS targeting DLPFC, combined with pain neuroscience education (PNE), significantly improved pain ratings, pain catastrophizing, and attention in patients with CLBP. They further inferred that tDCS has an activating and enhancing effect on PNE.

tDCS can also modulate abnormal neural pathways, improving postural control and pain in patients with CLBP. Both the direct and indirect motor pathways participate in postural control. Primary motor cortex M1 forms part of the direct motor pathway, while the supplementary motor area (SMA) and prefrontal cortex constitute components of the indirect motor pathway. The indirect motor pathway is typically activated during challenging tasks or when motor control is impaired (Herold et al., 2017). Li et al. (2023) found that patients with CLBP exhibited excessive activation in the motor cortex (M1) and DLPFC during challenging standing tasks, accompanied by poor postural stability. In a subsequent study, they investigated the effects of tDCS on postural control in patients with CLBP. Twenty CLBP patients were randomly assigned to either a tDCS group or a sham stimulation group, receiving 20 min of a-tDCS targeting M1 or sham stimulation, respectively. Results revealed significantly reduced activation in the left M1 region of the tDCS group, along with decreased functional connectivity between the left M1 and right DLPFC, and between the left M1 and bilateral frontal pole areas (FpA). This indicates that a-tDCS targeting M1 enhances neural processing efficiency, reduces compensatory demands on the sensorimotor neural network, and consequently improves postural control in patients (Wang et al., 2025). Other scholars suggest that tDCS may regulate pain transmission pathways to alleviate pain in patients with CLBP. Jiang et al. randomly assigned 60 patients with nonspecific CLBP to either a tDCS group or a sham treatment group. The tDCS group received stimulation targeting the primary motor cortex at an intensity of 20 mA. Results showed a significant reduction in pain intensity in the tDCS group. A potential mechanism involves downregulation of pain perception through top-down pain modulation pathways (Jiang et al., 2020).

## 3 Future outlook

### 3.1 Potential target for NIBS in treating CLBP: the cerebellum

Pain is a complex experience involving sensory, emotional, and cognitive factors. Recent studies have revealed that the cerebellum, in addition to its role in motor control, also plays a significant role in pain processing and emotional regulation (Manda et al., 2025). CLBP can impair cerebellar nociceptive responses, leading to heightened pain perception and impaired sensorimotor control. Zhang et al. (2025) investigated changes in functional connectivity within the anterior and posterior lobes of the cerebellum during CLBP. Twenty patients with CLBP and 18 healthy subjects underwent 3.0 T resting-state fMRI. Results revealed that patients with CLBP exhibited greater intrinsic connectivity between the left lobule V and the left insular cortex, left orbitofrontal cortex, and bilateral medial prefrontal cortex. Additionally, significant reductions were observed in connectivity between the right lobule V, bilateral Crus I, and the contralateral multimodal cerebral networks, such as the default mode network, salience network, and emotional network. As a key pathway for non-motor cerebellar functions, the fronto-cerebellar circuit participates in pain modulation and is also involved in pain-related cognitive and emotional processes. Additionally, the cerebellum plays a significant role in the regulation of pain anticipation. Pain anticipation is a complex state that influences pain perception, elicits abnormal sensations such as anxiety and fear, and affects cortical excitability and descending pain modulation pathways (Xu et al., 2024). The cerebellum participates in motor control, cognition, and emotional processing, potentially serving as a target for NIBS in the treatment of CLBP.

### 3.2 A potential NIBS technique for treating CLBP: transcranial focused ultrasound

In recent years, the therapeutic potential of transcranial focused ultrasound (tFUS) in chronic pain management has drawn scholarly attention (Staudt et al., 2025). tFUS is an emerging NIBS technique that utilizes acoustic energy to influence ion channels, synaptic transmission, and neural oscillations, thereby achieving precise modulation of specific brain regions (Shi et al., 2025). Compared with other NIBS techniques, tFUS can target both cortical and subcortical brain structures as well as deep brain structures to achieve therapeutic effects (Matt et al., 2024; Lee et al., 2024). Preliminary evidence suggests that tFUS may improve pain symptoms and emotional state in patients with chronic pain, demonstrating certain application potential (Shi and Wu, 2025). Low-intensity focused ultrasound (LIFU) possesses technical characteristics such as high spatial specificity and strong brain penetration. Research indicates that it can suppress pain by modulating pain-processing neural circuits in the brain (Kim et al., 2024). LIFUS promotes the release of neurotrophic factors, strengthens synaptic connections, and enhances brain plasticity (Issa et al., 2025). Research by Riis et al. (2024) has demonstrated that low-intensity focused ultrasound targeting the anterior cingulate cortex can rapidly and effectively alleviate pain in patients with chronic pain. LIFU targeting the cingulate cortex may represent a future direction for chronic pain management research (Strohman and Legon, 2025). Furthermore, regarding tFUS for pain treatment, further studies are needed on target site selection, stimulation parameter optimization, and dose optimization (Badran and Peng, 2024). Currently, there is a lack of published research on the use of tFUS for treating CLBP. Given the technical characteristics and mechanism of action of tFUS, it may potentially be applied to the treatment of CLBP in the future, and further research in this area is urgently needed.

## 4 Conclusion

The etiology of CLBP is complex. As the condition progresses, localized chronic pain in the lumbar region, alterations in sensory processing, and changes in movement patterns may lead to maladaptive changes in relevant brain regions. Changes in neural plasticity may be a key factor contributing to the protracted course and psychosomatic comorbidities observed in patients with CLBP. Thus, targeting brain regions to modulate neuroplasticity has emerged as a clinical approach for treating chronic low back pain. Research indicates that effective treatment can reverse anatomical and functional alterations in the brains of patients with CLBP (Seminowicz et al., 2011; Liu and Wan, 2025). Recent clinical practice has demonstrated that NIBS techniques can effectively alleviate both physical and psychological symptoms in patients with CLBP. When combined with other peripheral interventions, NIBS techniques offer unique advantages in improving pain and related symptoms among CLBP patients. Currently, rTMS and tDCS are widely used in the treatment of CLBP, while other NIBS technologies require further research and development. The primary motor cortex and prefrontal cortex are commonly selected targets for central treatment of CLBP. As neuropathological research continues to advance, other potential targets may be further identified. Enhancing cortical excitability, modulating pain-related neural networks, and suppressing neuroinflammation constitute the mechanism of action for NIBS in treating CLBP. Research on molecules and signaling pathways may become one of the key directions for future studies.

Although NIBS techniques show promising clinical potential for treating CLBP, many uncertainties remain that require further investigation. First, regarding indications, whether all cases of CLBP can benefit from NIBS treatment requires further research. A series of high-quality, large-scale, multicenter randomized controlled trials is urgently needed to provide evidence-based medical evidence, explore potential biomarkers and phenotypes, and establish a basis for central treatment strategies in patients with CLBP. Secondly, regarding treatment protocols, the selection of NIBS techniques and target sites, as well as the determination of stimulation intensity, frequency, and treatment duration for different CLBP patients, all require further refinement to achieve precise stimulation and personalized therapy (Lin et al., 2025; Mantovani et al., 2025). Finally, further research is needed to explore the benefits of combining NIBS techniques with other rehabilitation modalities and to establish a central-peripheral rehabilitation treatment system for CLBP. Preliminary clinical practice indicates that NIBS techniques targeting neural plasticity, when combined with peripheral interventions for CLBP, may produce additive or synergistic effects. Therefore, the “central-peripheral” treatment model also holds certain clinical application value in the management of CLBP. In summary, many aspects of NIBS treatment for CLBP require further research to better guide clinical application and benefit patients.

## Data Availability

The original contributions presented in the study are included in the article/supplementary material, further inquiries can be directed to the corresponding author.
